# A Kyber-Based Lightweight Cloud-Assisted Authentication Scheme for Medical IoT

**DOI:** 10.3390/s26072021

**Published:** 2026-03-24

**Authors:** He Yan, Zhenyu Wang, Liuming Lin, Jing Sun, Shuanggen Liu

**Affiliations:** School of Cyberspace Security, Xi’an University of Posts and Telecommunications, Xi’an 710121, China; 18922813121@stu.xupt.edu.cn (H.Y.); wzy13503922533@stu.xupt.edu.cn (Z.W.); linliuming@stu.xupt.edu.cn (L.L.); sunflower@stu.xupt.edu.cn (J.S.)

**Keywords:** medical Internet of Things (MIoT), post-quantum cryptography (PQC), Kyber algorithm, lightweight authentication, fuzzy commitment

## Abstract

The Medical Internet of Things (MIoT) has promoted smart healthcare through the deep integration of wearable devices, wireless communication, and cloud services. However, this framework faces security risks, as attackers may exploit public channels to impersonate legitimate devices or services and steal sensitive data. Therefore, establishing authentication between wearable devices and servers prior to data transmission is crucial. Existing schemes suffer from two critical drawbacks: vulnerability to quantum attacks and excessively high communication overhead, highlighting the need for improved solutions. The authors of this paper present a multi-factor identity authentication protocol to achieve post-quantum security and privacy protection. The scheme integrates lattice-based Kyber key encapsulation and a fuzzy commitment mechanism to secure biological templates and enable post-quantum key agreement. Additionally, hash functions and lightweight error correction codes are employed to reduce terminal communication overhead. The security of the scheme is rigorously proved in the Real-or-Random model, and the analysis confirms that the scheme satisfies common security requirements for wireless networks. The proposed scheme is also compared with existing schemes, and the results demonstrate that it achieves a balance between security and overhead.

## 1. Introduction

**Figure 1:**
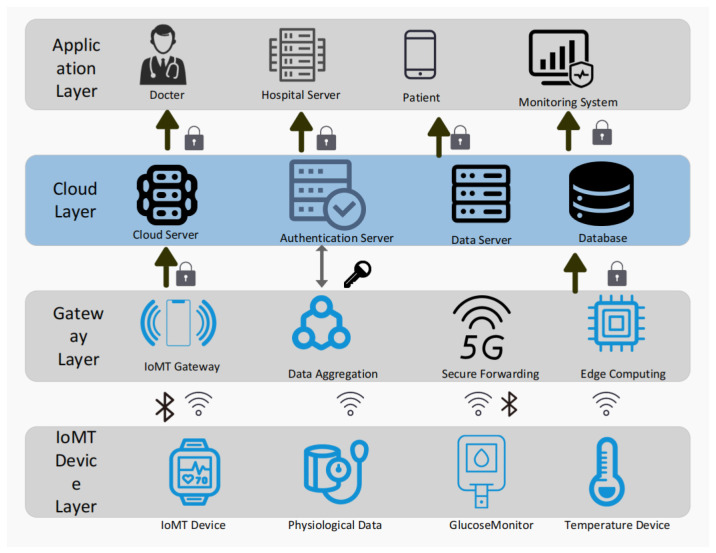
A typical cloud-assisted Medical Internet of Things architecture.

Advancements in 5G communication and intelligent sensing technologies have driven the development of the Medical Internet of Things (MIoT). By integrating healthcare scenarios with IoT technologies, MIoT constructs an interconnected ecosystem, thereby improving the efficiency of business processes and optimizing patient care services [1]. Figure 1 illustrates a typical cloud-assisted MIoT architecture. However, MIoT devices generally feature limited computing resources and open deployment environments. The transmitted data, such as physiological signs and medical records, contains a wealth of sensitive information. Once vulnerabilities exist in the authentication phase, security incidents such as data leakage, forgery, or tampering are likely to occur, posing serious threats to patient privacy and life safety [2].

As the first line of defense for MIoT communication security, authentication mechanisms must simultaneously satisfy the requirements of identity legality verification, key agreement security, and lightweight deployment. Mainstream authentication schemes are typically constructed based on traditional cryptography, such as bilinear pairing or Elliptic Curve Cryptography (ECC) [3,4], which can provide a certain level of security under classical computing conditions. The emergence of Shor’s algorithm, detailed in [5,6], poses a fundamental threat to classical cryptography by efficiently solving integer factorization and discrete logarithms. Therefore, existing cryptographic systems relying on these hardness assumptions are facing “quantum threats” [7]. In addition, traditional algorithms incur high computational and communication overheads, making it difficult to adapt to the limited computing power and energy reserves of MIoT terminals [8,9,10,11], leading to increased authentication latency and reduced device battery life [12].

The proposal of post-quantum cryptography (PQC) algorithms provides a new approach to addressing the aforementioned issues. Kyber is one of the first post-quantum standardized key encapsulation mechanisms (key encapsulation mechanism (KEM)) determined by NIST, offering dual advantages of quantum security and efficient implementation. It is fundamentally built on the Module Learning With Errors (Module-LWE) problem. While maintaining strong security, it offers short key and ciphertext sizes and low computational complexity, which is well-suited to resource-constrained environments [13].

In view of this, this paper focuses on the authentication requirements in MIoT environments and proposes a lightweight authentication scheme based on Kyber. By combining post-quantum cryptographic techniques with lightweight design principles, the scheme provides resistance to quantum attacks while alleviating computational and communication overhead at the device side. It provides reliable and efficient identity authentication and key agreement services for MIoT terminals, aiming to offer technical support for the secure development of smart healthcare.

The structure of the remaining part of this article is as follows: Section 2 reviews and summarizes the related research work, with a focus on introducing the current development status of traditional identity authentication schemes and post-quantum cryptosystems; Section 3 introduces the necessary basic knowledge for the scheme design, including the Kyber key encapsulation mechanism, fuzzy commitment technology, and system model; Section 4 details the proposed lightweight multi-factor authentication and key agreement scheme, covering the system initialization, user registration, and authentication and session key establishment stages; Section 5 evaluates the security characteristics of the protocol from the aspects of formal proof and security analysis; Section 6 presents the performance analysis results and compares them with existing schemes in terms of computational and communication costs; Section 7 summarizes the entire article and provides conclusions.

## 2. Related Work

As Medical Internet of Things (MIoT) systems and intelligent sensing technologies continue to evolve, secure authentication for resource-constrained devices has become an increasingly critical requirement. Traditional authentication schemes mostly rely on classical cryptography (such as ECC and RSA), which, although providing a certain degree of security, are difficult to resist quantum computing attacks and often fail to meet lightweight deployment requirements due to high computational and communication overheads. In recent years, post-quantum cryptography (PQC) and lightweight design technologies have emerged as research hotspots. This section reviews related work from two dimensions: security enhancement and overhead optimization, and points out the innovations of the proposed scheme.

### 2.1. Traditional Authentication Schemes and Their Limitations

Elliptic Curve Cryptography (ECC)-based authentication schemes are commonly adopted in IoT settings, offering short key sizes and high computational efficiency. A lightweight authentication approach based on ECC was introduced in [14], where dynamic keys are generated by integrating one-time passwords (OTPs) with isogeny, thereby mitigating the risks associated with long-term pre-shared key (PSK) usage. The scheme achieves microsecond-level authentication latency on ordinary devices (such as laptops), but the ECC algorithm it relies on still faces quantum computing threats (such as Shor’s algorithm). In addition, the computational overhead of the scheme increases significantly on resource-constrained devices (such as Raspberry Pi; OTP generation takes approximately 1130 μs), and no formal security proof is provided.

Another type of lightweight design mainly relies on symmetric primitives such as hash and XOR to reduce implementation costs and transmission burdens. The scheme by Gupta et al. [15] performs well in overhead control but fails to meet the requirement of forward security and is premised on pre-shared keys, thus having limited resistance to key leakage attacks. The multi-factor authentication scheme by Sahoo et al. [16] improves security by combining biometrics with passwords, but its ECC foundation still cannot resist quantum attacks, and the scheme involves multiple communication rounds, increasing transmission overhead.

In high-density MIoT environments, communication efficiency can also be improved by optimized reader anti-collision mechanisms. For example, distributed parallel particle swarm optimization-based RFID anti-collision methods have been shown to reduce identification latency for multiple devices [17]. Such communication-layer optimizations can further enhance the performance of the proposed authentication scheme.

Overall, traditional schemes have made certain progress in lightweight design, but there are still deficiencies in the balance between security (especially quantum resistance) and overhead.

### 2.2. Research Progress of Post-Quantum Cryptography (PQC) in Authentication

To cope with the challenges posed by quantum computing, lattice-based authentication schemes have emerged as a dominant research direction [6]. In this context, the Module Learning With Errors (MLWE) problem has been extensively adopted for key exchange and authentication, owing to its robustness and parameter flexibility. Dabra et al. proposed LBA-PAKE [18], which realizes anonymous password authentication based on the Ring-LWE (RLWE) assumption and formally proves the protocol security using the ROR model. The scheme supports forward security and key reuse but relies on an error reconciliation mechanism, resulting in high computational complexity (server-side overhead reaches the millisecond level) and failure to effectively solve the Signal Leakage Attack (SLA) problem.

In the PQC standardization process, the Kyber algorithm, as the KEM selected by NIST [19], has attracted much attention due to its small key and ciphertext sizes. Chen et al. combined Kyber with fuzzy commitment [16] to design a multi-factor authentication scheme, which improves the reliability of identity verification through biometric binding and proves the indistinguishability of session keys based on the ROR model. In addition, recent work has applied Kyber KEM and fuzzy extractor-like technologies to multi-server MFA architectures and provided formal proofs under the ROR model, but there are still issues of communication and terminal computational burdens [20]. However, the MLWE operations (such as vector multiplication) of this scheme on terminal devices still generate high computational loads [10], and the communication data packet size is relatively large (approximately 10,016 bits), which is not conducive to low-bandwidth networks.

### 2.3. Lightweight Design Technologies and Overhead Optimization

To reduce overhead, researchers have made efforts in both algorithm optimization and protocol structure:

Error Reconciliation Mechanism: Ding et al. used modular arithmetic and signal functions (such as Cha(), Mod2()) to reduce the data transmission volume in key agreement [18], but multiple polynomial operations are required, increasing the computational burden on terminals. Saliba et al. proposed a reconciliation technology based on Voronoi cells [16], which reduces the failure probability but is complex to implement and difficult to adapt to low-storage devices.

Fuzzy commitment and Biometric Template Protection: By binding biometrics with Hamming codes and fuzzy commitments, template storage is avoided, and storage overhead is reduced. However, the decoding process requires error correction calculations, which may introduce additional latency. In addition, biometric template protection systems for IoT scenarios have been systematically studied in recent years, emphasizing robustness and transferable applications under key leakage and privacy constraints [21].

Round Reduction and Message Compression: Abdaoui et al. combined ECC with fuzzy logic to generate random numbers [16], reducing the number of authentication rounds, but the scheme does not consider multi-factor integration and has weak security. The scheme in [16] achieves batch verification through timestamps and random numbers, reducing the number of interactions, but has strict requirements on time synchronization and is vulnerable to replay attacks.

Despite the attempts to optimize overhead in the above work, the balance between computation and communication is still unsatisfactory. As an illustration, the LBA-PAKE scheme reported in [18] incurs a bandwidth cost of up to 13,376 bits, whereas the ECC-based approach in [14], despite its lower bandwidth consumption, fails to provide quantum-resistant security. Table 1 presents a comparison of the related work on the IoMT certification scheme.

### 2.4. Research Gaps and Contributions of This Paper

A comprehensive analysis of the existing work shows that there are mainly shortcomings such as an imbalance between security and cost, insufficient integration of multiple factors, and poor resource adaptation [22]. Aiming at the above problems, this paper proposes a lightweight multi-factor authentication protocol based on Kyber. The trade-off between security and overhead is achieved through the following innovations:

Lightweight Post-Quantum Foundation: By adopting the Kyber512 algorithm and combining the fuzzy commitment mechanism with the SHA-3 hash function, while ensuring quantum security, an efficient key compression is achieved, controlling the size of the core key to 512 bits. This effectively reduces storage and computing costs. Here, “512-bit compressed key” refers to the compact storage mechanism on the MIoT device side. The device does not need to save the complete 800-byte Kyber public key, but only stores the 32-byte seed used to generate the public key components and the 32-byte hash value h=H(k) for biometric verification. This reduces the total storage requirement of the core security credential to 512 bits, thus meeting the application requirements of resource-constrained medical devices.

Overhead Optimization Mechanism: A one-way authentication chain and timestamp batch verification are designed to reduce the number of interactions and communication volume (reducing bandwidth by 20% compared with [4]); terminals only need lightweight operations (such as XOR and hash), adapting to resource-constrained devices.

Multi-Factor Security Enhancement: Joint binding of biometrics and passwords is introduced. Fuzzy commitment is used to prevent template leakage, supporting forward security and anonymity, and resisting man-in-the-middle, replay, and other attacks.

Under the premise of maintaining PQC security strength, the proposed scheme significantly optimizes overhead efficiency, providing a more practical lightweight authentication solution for MIoT environments.

## 3. Preliminary Knowledge

### 3.1. Kyber Key Encapsulation Mechanism

Kyber was selected as a post-quantum key encapsulation scheme in the NIST standardization effort. Built upon module lattice-based cryptographic assumptions, it provides chosen-ciphertext security within the random oracle framework. The scheme supports three parameter sets, namely Kyber512, Kyber768, and Kyber1024, which align with NIST security levels 1, 3, and 5 and offer security strength comparable to AES-128, AES-192, and AES-256, respectively. The parameter n = 256 (balancing security and the extensibility of k), and k is a multiple of n used to extend security. Specifically, Kyber512 offers a quantum security strength of approximately $2^{118}$ operations based on the Core-SVP (Shortest Vector Problem) metric. Its security level is comparable to that of AES-128 and can withstand attacks from quantum attackers.

In this study, we selected the Kyber-512 parameter set (k = 2) to achieve a balance between post-quantum security and resource consumption. Although parameter sets with higher security levels, such as Kyber-768 and Kyber-1024, can provide greater security margins, they usually come with higher computational and communication costs, often increasing system latency and energy consumption by approximately 30–50%, thereby adversely affecting the battery life of resource-constrained IoT sensor batteries. In contrast, Kyber-512 meets the requirements of Security Category 1 in the NIST post-quantum cryptography standard and can provide a quantum security strength equivalent to AES-128. This security level ensures the long-term confidentiality of medical data while maintaining the real-time performance required by wearable devices, making it more suitable for application in resource-constrained medical IoT environments.

Algorithms 1–3 [13,23] describe the key generation, encapsulation, and decapsulation procedures of Kyber.CCAKEM.
**Algorithm 1** Kyber.CCAKEM.KeyGen().**Output**: Public key $\mathrm{pk}\in B^{12\cdot k\cdot n/8+32}$ **Output**: Secret key $\mathrm{sk}\in B^{24\cdot k\cdot n/8+96}$
1: $z\leftarrow B^{32}$
2: $\left(\mathrm{pk},{\mathrm{sk}}^{\prime }\right):=\mathrm{Kyber}.\mathrm{CPAPKE}.\mathrm{KeyGen}\left(\right)$
3: $\mathrm{sk}:=({\mathrm{sk}}^{\prime }\|\mathrm{pk}\|H\left(\mathrm{pk}\right)\|z)$
4: **return** (pk,sk)


During key generation, a 32-bit random string *z* is sampled, after which KYBER.CPAPKE.KeyGen is invoked to derive the public component pk and a secret component $sk^{\prime }$. The final secret key is then constructed from $sk^{\prime }$, pk, H(pk), and *z*, and the resulting key pair (pk,sk) is output.
**Algorithm 2** Kyber.CCAKEM.Enc(pk).**Input**: Public key $\mathrm{pk}\in B^{12\cdot k\cdot n/8+32}$ **Output**: Ciphertext $c\in B^{d_{u}\cdot k\cdot n/8+d_{v}\cdot n/8}$ **Output**: Shared key $K\in B^{*}$
1: $m\leftarrow B^{32}$
2: $\left(\vec{K},r\right):=G\left(m\|H\left(\mathrm{pk}\right)\right)$
3: c:=Kyber.CPAPKE.Enc(pk,m,r)
4: $K:=\mathrm{KDF}(\vec{K}\|H\left(c\right))$
5: **return** (c,K)


During the key encapsulation stage, Algorithm 2 first samples a 32-byte random string *m*. Subsequently, it directly derives the intermediate key $\vec{K}$ and the random number *r* using *m* and the public key hash, that is, $\left(\vec{K},r\right)=G(m||H\left(pk\right))$. Then, it calls Kyber.CPAPKE.Enc() to generate the ciphertext *c* using *m* and *r*. Finally, it binds the intermediate key and the ciphertext hash through $KDF(\vec{K}||H\left(c\right))$ to output the final shared key K.
**Algorithm 3** Kyber.CCAKEM.Dec(c, sk)**Input**: Ciphertext $c\in B^{d_{u}\cdot k\cdot n/8+d_{v}\cdot n/8}$ **Input**: Secret key $\mathrm{sk}\in B^{24\cdot k\cdot n/8+96}$ **Output**: Shared key $K\in B^{*}$
1: (s,pk,h,z):=sk
2: $m^{\prime }:=\mathrm{Kyber}.\mathrm{CPAPKE}.\mathrm{Dec}\left(s,u,v\right)$
3: $\left(\vec{K},r^{\prime }\right):=G\left(m^{\prime }\|h\right)$
4: $c^{\prime }:=\mathrm{Kyber}.\mathrm{CPAPKE}.\mathrm{Enc}\left(\mathrm{pk},m^{\prime },r^{\prime }\right)$
5: **if** $c=c^{\prime }$ **then**
6:     **return** $K:=\mathrm{KDF}(\vec{K}\|H\left(c\right))$
7: **else**
8:     **return** K:=KDF(z‖H(c))
9: **end if**
10: **return** K


During the key decryption stage, Algorithm 3 first extracts the private key components by parsing (s,pk,h,z):=sk. Subsequently, it decrypts the ciphertext *c* using *s* to obtain the plaintext $m^{\prime }$. Then, it derives the intermediate key ${\vec{K}}^{\prime }$ and the random number $r^{\prime }$ through $G(m^{\prime }||h)$, and re-encrypts to generate the verification ciphertext $c^{\prime }$. If *c* = $c^{\prime }$, the shared key *K* derived from ${\vec{K}}^{\prime }$ is returned; otherwise, a pseudo-random key is returned using the random seed *z*, ensuring the robustness of the protocol against adaptive chosen-ciphertext attack (CCA).

During decapsulation, the ciphertext *c* together with the secret key sk is processed to recover pk, *h* and *z*. The plaintext $m^{\prime }$ is then derived via the CPAPKE decryption procedure, after which $G(m^{\prime }||h)$ is applied to obtain $\left(K^{\prime },r^{\prime }\right)$. A verification ciphertext $c^{\prime }$ is subsequently reconstructed. If $c=c^{\prime }$, the session key is computed as $KDF(K^{\prime }||H\left(c\right))$; otherwise, the output key is generated as KDF(z||H(c)).

In the standard Kyber512 specification, the byte length of the public key pk is 12·k·n/8+32, mainly consisting of the coefficient sequence of the polynomial vector *t*. In this protocol, we have changed the traditional representation form of the ciphertext and the public key. Instead of storing or transmitting the complete coefficient vector, we represent the vector by transmitting a 256-bit random seed. According to the design principle of Kyber, the vector *t* is essentially the result of the operation of the matrix *A* generated by the seed through an expandable output function (XOF) and the private key vector. Therefore, transmitting the seed is mathematically equivalent to transmitting the complete public key, but it achieves an extremely high compression ratio in the communication representation.

### 3.2. Fuzzy Commitment

Fuzzy commitment is a new cryptographic primitive combining error-correcting codes and cryptography [24,25], used to solve the secure commitment problem under information errors, and is suitable for fields such as biometric authentication.

The core logical structure is as follows: Let $bio\in {\left\{0,1\right\}}^{n}$ be the user’s original biometric feature vector, and *k* be the persistent secret value pre-selected during the registration stage, which serves as a static anchor point for user authentication and remains unchanged after registration. The system calculates the auxiliary data δ=bio⊕ECC(k) and publicly stores it, where ECC(·) is the error-correcting code encoding function. At the same time, the hash value h=H(k) is stored for subsequent verification. During the authentication stage, the user provides the biometric feature with noise bio′, and calculates $c^{\prime }=bio^{\prime }⊕\delta$ and attempts to restore k′ = f(c′) using the error-correcting code decoding function f(·). If $H(k^{\prime })=h$, it proves that the Hamming distance between bio′ and bio is within the error correction range, and the commitment is successfully opened.

In the context of MIoT applications, the disclosure of auxiliary data δ will lead to the loss of biometric entropy. To evaluate the security of the scheme, we introduce effective entropy (Effective Entropy) for quantitative analysis. Let the entropy of the original biometric template be H(bio), and the error correction code parameters be (n,κ,2t+1), then the effective entropy $H_{eff}$ is defined as:

$$H_{eff}=H\left(bio\right)-\left(n-\kappa \right)$$

Taking the commonly used (255,131,18) Lightweight error-correcting code in the MIoT environment as an example, the introduced information leakage (redundancy) is 255−131=124 bits. Assuming the original template entropy H(bio)=256 bits, the remaining effective entropy $H_{eff}=132$ bits. This result indicates that even after considering the entropy loss caused by the error correction code, the system can still provide a security strength of over 128 bits, which is sufficient to resist brute-force attacks in the post-quantum era and meets the strict privacy compliance requirements in the medical IoT environment. Here, the assumption that H(bio)=256 bits is based on empirical research on high-dimensional biological features. For instance, templates based on the iris have been proven to provide more than 240 bits of freedom [26], while the fusion of multimodal biological features or the extraction of deep learning features based on physiological signals (such as electrocardiogram or pulse wave spectroscopy) can always generate an entropy value of more than 200 bits [27]. It should be noted that fuzzy commitment may have template leakage risks under certain conditions, so the system design should enhance security by combining parameter selection and entropy assumptions [28,29].

### 3.3. System Model

**Figure 2:**
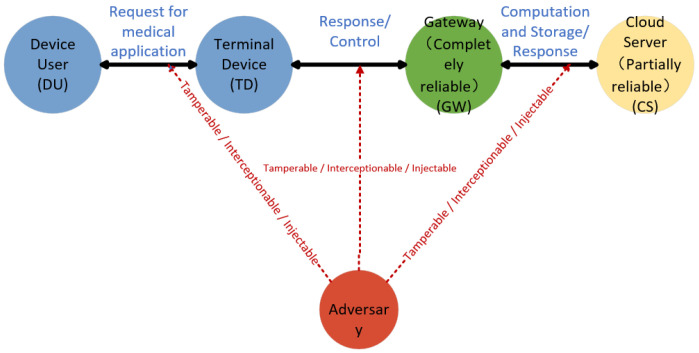
System model.

This section provides an overview of the system framework relied upon by the proposed mechanism. The framework consists of three core roles: device users (DU), terminal device (TD), and cloud servers (CS). Device users (DU) refer to individuals who wish to access medical applications through edge terminals; terminal device (TD) generally refer to any wearable or implantable intelligent medical devices that can connect to the cloud server and are vulnerable to physical acquisition and side-channel attacks in the public domain; cloud servers (CS) not only provide medical computing and storage but also undertake the responsibilities of network initialization and key distribution. Figure 2 illustrates the overall model architecture.

The model set forth in this article is as follows:

1. The CS node is officially designated as an honest but curious entity, and its behavior follows the following precise assumptions: 1. CS strictly follows the prescribed protocol algorithm execution process and will not maliciously deviate from or tamper with the protocol logic. 2. CS will attempt to infer the user’s sensitive information from the stored credentials or the received authentication records. 3. It will not collude with external rivals. Moreover, the storage of CS is assumed to be vulnerable to external attacks.

2. The gateway is assumed to be a trusted third party because it is deployed in a controlled environment of the medical institution and is protected by enterprise-level firewalls and physical access controls. In the attacker model, the adversary can perform data tampering, blocking or deletion on the public communication channels, and has the ability to forge any message to inject into the network flow.

3. The adversary has comprehensive knowledge of the system’s public parameters and the protocol operation mechanism, but their access capabilities are limited, and they are unable to concurrently obtain the complete set of three elements: the user password, biometric data, and the secret information stored in the smart card.

## 4. The Proposed Scheme

This part will detail our proposed scheme from four aspects: initialization, registration, authentication and key agreement.Table 2 illustrates the notations used in the following text.

### 4.1. Initialization

Before the server can communicate with the user, the server needs to complete the following steps to initialize:

1. A positive integer *n* is selected, and a prime *q* and a positive integer *k* are randomly selected.

2. Set the polynomial ring $R=Z_{q}\left[X\right]/\left(X^{n}+1\right)$, and generate a random matrix $A\in R_{q}^{k\times k}$. Define the central binomial distribution $\beta_{\eta }$ as the sampling source for the error term and the secret vector, which is used to define the $\chi^{k}$ distribution range of MLWE.

3. Select $s,e\in R_{q}^{k}$ and calculate t=As+emodq, then the public key is mpk=(A,t), the private key msk=s, and mpk is distributed to all terminals. The system master public key mpk defined in this article is directly equivalent to the public key pk=(A,t) of Kyber, where t=As+e; the system master private key msk corresponds to the private key sk of Kyber.

4. Select a secure hash function H(.).

5. Choose lightweight coding schemes for error recovery, for example, Hamming or reduced-complexity BCH variants, that are able to correct up to t errors.

6. Biometric data is quantized as a binary vector $bio\in {\left\{0,1\right\}}^{n}$.

7. The random key $k\in {\left\{0,1\right\}}^{m},m=128$.

### 4.2. Registration

To obtain access to the server, a new user must complete the registration process in the manner described below.

1. The user inputs $ID_{i}$, password $PWD_{i}$, and biometric information $BIO_{i}$ at the terminal. The $BIO_{i}$ is quantized into a binary vector $bio\in {\left\{0,1\right\}}^{n}$, and a lightweight error correcting code is used to encode *k*. Generated code word $c_{i}=Encode\left(k\right)\in {\left\{0,1\right\}}^{256}$, calculating secondary data $\delta_{i}=c_{i}⊕bio_{i}$, hash h=h(k), generates a random string $C_{i}=H\left(\delta_{i}mpk\right)$.

2. The temporary identity $TID_{i}=H(C_{i}||ID_{i})$ and temporary password $TPW_{i}=H(ID_{i}||PWD_{i})$ are calculated, and the terminal sends ${TID}_{i}$ and ${TPW}_{i}$ to the server

3. The server selects $ID_{j}$ and calculates $TID_{j}=H(ID_{j}\left||msk\right)$, $TID_{i}^{*}=H(TID_{i}\left||msk\right)$, $TPW_{i}^{*}=TID_{i}^{*}⊕TPW_{i}$. The parameters ${TID}_{j}$ and $TPW_{i}^{*}$ are subsequently delivered to the TD over a secure link.

4. The terminal recovers $TID_{i}^{*}$ by XOR operation, and $TID_{i}^{*}=TPW_{i}^{*}⊕TPW_{i}$ The terminal then calculates $V2=H(ID_{i}||PWD_{i}||C_{i}||mpk)$.

5. Terminal storage V2, $TID_{j}$, h=H(k), $\delta_{i}$ and server storage $TID_{i}^{*}$, ${TPW}_{i}$, $ID_{j}$.

The security channel mentioned at this stage refers to a temporary, out-of-band communication link that is only used during the one-time registration process of the terminals. After registration, all subsequent authentication and key negotiation stages are conducted over an insecure public channel.

**Figure 3:**
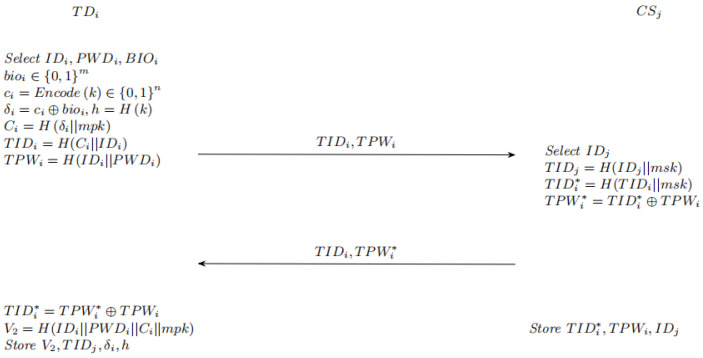
Registration phase.

The process is shown in Figure 3.

### 4.3. Authentication and Key Agreement

This section describes how the terminal and the server validate each other and establish a session key to support later data exchanges.

1. The terminal sends a simple request to the server, and the server randomly selects a secret number $R_{j}$.

2. Th terminal inputs ID, password PWD, and biometric information BIO. The biometric BIO is quantized into a binary vector $bio\in {\left\{0,1\right\}}^{n}$, and then $c^{\prime }=bio⊕\delta$ is computed, decoded ($c^{\prime }$) using Hamming code, and the secret key $k^{\prime }=Decode\left(c^{\prime }\right)$ is recovered. Verify whether $H(k^{\prime })$ and *h* are equal; if so, $k^{\prime }=k$ and generate $C^{\prime }=H\left(\delta ||mpk\right)$; if not, terminate the session. Then we compute $V2^{\prime }=H\left(ID||PWD||C’||mpk\right)$. $V2^{\prime }$ is compared with V2, if it is not the same, the conversation is terminated; otherwise, the authentication passes and $P=V2^{\prime }⊕H(ID||PWD||C^{\prime })$ is calculated. Randomly select vectors $s_{i},e_{i}\leftarrow \chi^{k}$, calculate $t_{i}=A\cdot s_{i}+e_{i}$,$pk=\left(A,t_{i}\right)$. The $e2=CSPRNG(seed=r_{i}||ID||t1,\chi^{k})$ is calculated using the pseudo random number generator, and then calculate the $Auth=H(ID||PWD||P||TID_{j}||t1||v||R_{j})$, $v=t_{i}⊕r_{i}+e2+H\left(ID||t1\right)$. The server then uses the SHAKE-128 XOF to reconstruct the required group parameters based on this seed. This seed-based mechanism is a key factor in achieving lightweight communication. $t_{i}\to seed\_t_{i}$,$r_{i}\to seed\_r_{i}$, compress ti and $r_{i}$, and the terminal sends $seed\_t_{i}$, $seed\_r_{i}$, timestamp t1, Auth to the server over a secure channel.

3. Upon obtaining the transmitted data from the terminal, an initial check is performed to verify whether the condition $\left|t2-t1\right|<T$ (representing the maximum allowable transmission delay) holds. If the verification fails, the conversation is abandoned; otherwise, $t_{i}$ and $r_{i}$ are resumed. To ensure that this seed-based representation can be correctly parsed, the recipient (the server) needs to perform a reconfiguration process. This process is consistent with the standard Kyber parameter generation logic, that is, using SHAKE-128 to deterministically expand the seed: $t_{i}=\mathrm{SHAKE}-128\left(seed\_t_{i}\|\mathrm{index},6400\right)$ Here, the 6400-bit output length precisely corresponds to the coefficient representation scale of the vector $\vec{t}$ in Kyber512. This seed-based ciphertext representation method (ciphertext representation) compresses the originally lengthy polynomial data into a compact hash input, significantly reducing the bandwidth burden on the MIoT terminal. In order to ensure that the compression process does not introduce exploitable correlations, the reconstruction process introduces a position index as a domain separator. $t_{i}=SHAKE-128(seed\_t_{i}\left||index,6400\right)$, $r_{i}=SHAKE-128(seed\_r_{i}\left||index,6400\right)$, and e2’ and v’ are calculated. Then, $Auth^{*}=H(id||pwd||P||TID_{j}\left||t1||v’|\right|R_{j})$ is computed. If Auth is not equal to $Auth^{*}$, the authentication fails, and the conversation is aborted; otherwise, the authentication passes. Random vector $s_{j},e_{j},e_{j}^{\prime }\leftarrow \chi^{k}$, calculate $t_{j}=A\cdot s_{j}+e_{j}$, $u_{j}=A^{T}\cdot r_{j}+e_{j}^{\prime }$, $pk’=(A,t_{j})$. Then, calculate $tsk=KEM(u_{j},msk)$, the key generation algorithm to calculate the key $SK=HKDF(tsk,salt=TID^{*}||pk||pk’)$, finally calculated as $Auth1=H(TID_{i}^{*}||pk||pk’||tsk||SK||t3)$. $t_{j}$ and $r_{j}$ are compressed $t_{j}\to seed\_t_{j}$,$r_{j}\to seed\_r_{j}$, and the server sends Auth1, t3, $seed\_t_{j}$, $seed\_r_{j}$ to the terminal.

4. After receiving the information sent by the server, the terminal first determines whether t4−t3<T (maximum transmission delay) is satisfied. If the verification fails, it will give up the conversation; otherwise, $t_{j}$ and $r_{j}$ will be resumed. $t_{j}=SHAKE-128(seed\_t_{j}\left||index,6400\right)$, $r_{j}=SHAKE-128(seed\_r_{j}\left||index,6400\right)$, Then $tsk=KEM(u_{j},msk)$ and $SK=HKDF(tsk,salt=TID_{i}^{*}||pk||pk’)$ are calculated and $Auth1^{*}$ is calculated. Compare $Auth1^{*}$ and Auth1 to see if they are equal. If they are equal, then both parties store SK; otherwise, give up the conversation. After the server calculates the session key SK, it must immediately erase the temporary random vectors $s_{j}$ and $e_{j}$ used for this round of calculation from the memory. This mechanism ensures that the subsequent leakage of msk will not lead to the re-decoding of the historical session.

**Figure 4:**
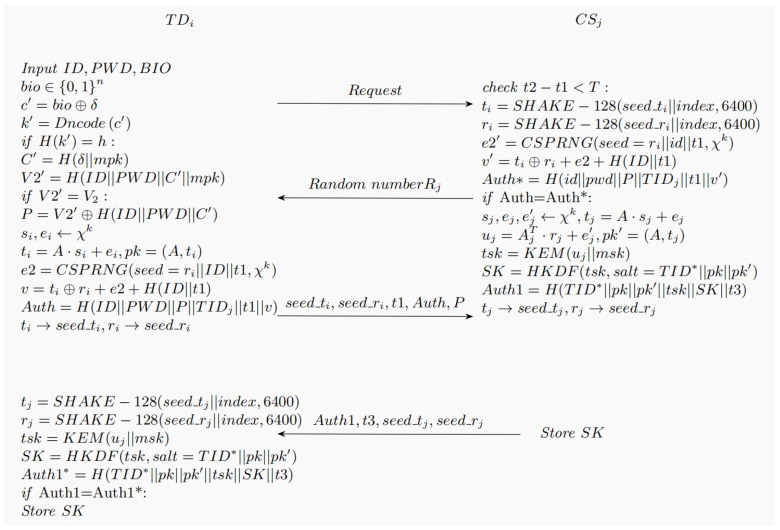
Mutual authentication and session key establishment.

The process is shown in Figure 4.

## 5. Security Analysis

This section systematically analyzes and illustrates the protection capabilities of the scheme by adopting two complementary approaches: formal proof and informal argument.

### 5.1. Formal Analysis

Within the Real-or-Random (ROR) security framework, formal validation confirms that the developed MIoT authentication protocol exhibits session key indistinguishability in a post-quantum setting.

Let the set of sessions be denoted Γ, and the set of participants be denoted *P*, and be partitioned into two nonempty subsets: clients u∈P and servers s∈P. For any participant P∈P, denote by $\Gamma_{i}^{P}$ its ith protocol instance (random oracle interface), and all instances share the common parameter mpk (generated and issued by the challenger *C*).

The attacker *A*, a probabilistic polynomial-time algorithm, obtains information by submitting a polynomial number of queries to the random oracle Γ and observing the responses.

The challenger *C* first generates the public parameters params and returns them to *A*. *A* can then invoke the following oracle query:

Setup $\left(\Gamma_{i}^{P},m\right)$: *C* returns the master key msk with the global parameter params.

Execute $\left(\Gamma_{i}^{\mu },\Gamma_{j}^{\varsigma }\right)$: The ith client instance $\Gamma_{i}^{\mu }\mu$ executes a complete protocol session with the JTH server instance $\Gamma_{j}^{\varsigma }$ A gets all the interaction transcripts.

Send $\left(\Gamma_{i}^{P}\right)$: Sends a message *m* to the actor instance $\Gamma_{i}^{P}$. This instance updates the state according to the protocol specification and returns a possible response message $m^{\prime }$. This query allows *A* to inject, modify, or replay messages proactively.

Corrupt (P): Returns the long-term stored parameters (msk,V2,δ,h,etc.) of participant P∈P. Corruption does not leak the bio measurements of the current session, only the stored state (C,δ).

Reveal$SK\;(\Gamma_{i}^{P})$: This returns the computed session key $\Gamma_{i}^{P}$ and k for the instance $\Gamma_{i}^{P}$.

Test $\left(\Gamma_{i}^{P}\right)$: Only available if $\Gamma_{i}^{P}$ is a “clean” session. An instance is considered fresh if it has reached the Accepted state, the adversary has not issued a RevealSK query on the instance or its partner, and no Corrupt query occurs on the involved participants during the active phase of the session.

Each instance $\left(\Gamma_{i}^{P}\right)$ maintains a variable set $V=\left\{state,acc,term,sid,pid,sk,b\right\}$, where $state\in \left\{Accepted,Rejected,Active\right\}$

Under the ROR model, the security of the proposed AKE protocol reduces to the ability of attacker *A* to distinguish the uniform random string from the real session key SK. *A* is given the oracle set Γ=(Execute,Send,Corrupt,RevealSK,Test) and can activate q_s sessions and initiate q_h hash queries at most in polynomial time. The ultimate goal of attacker *A* is to correctly determine the string returned by the Test query. Let *b* be the random bit hidden by the challenger: the true SK is returned for b=1, and a uniform random string of equal length is returned for b=0. Let $b^{\prime }$ be the final output of *A*, then the success probability of *A* can be naturally expressed as

(1) $$|Pr\left[b^{\prime }=1|b=1\right]-Pr\left[b^{\prime }=1|b=0\right]|.$$

This absolute value is the measure by which *A* distinguishes the real key from the random string, so the ROR advantage is defined as follows:

(2) $$Adv_{ROR}\left(A\right)=\left|Pr\left[b^{\prime }=1|reality\right]-Pr\left[b^{\prime }=1|random\right]\right|.$$

If Adv_ROR(A) is a negligible function negl(λ), the protocol is said to satisfy session key indistinct under the ROR model.

Assumption of Difficulty

A1. Kyber-IND-CPA: The ciphertext of Kyber512 is indistinguishable from a random string.

A2. MLWE Assumption: We assume the hardness of the Module-LWE problem. Let n,k,q be integers and χ be a distribution over $R_{q}$. The problem is to distinguish between the MLWE distribution $L_{MLWE}=\left\{\left(A,As+e\right)\right\}$ and the random distribution $L_{Random}=\left\{\left(A,t\right)\right\}$. The advantage of an adversary A is defined as $Adv_{MLWE}\left(A\right)=\left|\mathrm{Pr}\left[A\left(L_{MLWE}\right)=1\right]-\mathrm{Pr}\left[A\left(L_{Random}\right)=1\right]\right|$. This advantage is assumed to be negligible for any polynomial-time adversary.

A3. Collision-resistant hashing: SHA3-256 has a negligible collision probability with SHAKE128.

A4. Fuzzy commitment security: If the minimum entropy of the biometric bio≥α and the Hamming code error correction failure probability $\leq 2^{-\lambda }$, then the attacker has a negligible probability of recovering the correct *k* from commitment (C,δ).

A5. XOF Random Oracle Assumption: Assume that SHAKE-128 is used as an extensible output function (XOF) operating under the random oracle model. For any different input seeds or domain separators, the generated output sequence is computationally pseudo-random and mutually independent, which ensures that the vector reconstructed from $seed_{t_{i}},seed_{r_{i}}$ fully inherits the hardness attribute of the MLWE problem.

**Theorem** **1.** *Suppose that attacker A activates at most q_s sessions and initiates q_h hash queries; then, there is*

(3) $$\begin{array}{cc} {\mathrm{Adv}}_{\mathrm{ROR}}(A) & \leq 2\cdot q_{s}\cdot {\mathrm{Adv}}_{\mathrm{Kyber}-\mathrm{IND}-\mathrm{CPA}}\\ \; & \;+2\cdot q_{s}\cdot {\mathrm{Adv}}_{\mathrm{MLWE}}\\ \; & \;+q_{s}\cdot {\mathrm{Adv}}_{\mathrm{Hash}}\\ \; & \;+q_{s}\cdot {\mathrm{Adv}}_{\mathrm{Fuzzy}}\\ \; & \;+\frac{q_{h}^{2}+q_{s}^{2}}{2^{\lambda }} \end{array}$$

**Proof** **of Theorem 1.** At this point, the experiment is consistent with the real operation of the protocol. The probability of success of attacker A in Game0 is denoted as Pr[S0]. By definition, there is

$$|Pr\left[S0\right]-\frac{1}{2}|=\frac{1}{2}Adv_{ROR}\left(A\right)$$

□

Game 1 (excludes hash collisions)

The game directly takes into account the collision resistance of the hash values transmitted during the mutual authentication interaction (such as $TID_{i}$, Auth and Auth1). Since SHA-3 has extremely high collision resistance, the probability that an attacker can pass the verification by forging these specific message sequences is limited within the birthday attack boundary.

If any hash output collision (such as H(k), H(δ||mpk), Auth, Auth1, etc.) occurs during the experiment, the experiment will be stopped immediately.

Since the SHA 3 series satisfies collision resistance, the collision probability is given by the birthday bound, which satisfies:

(4) $$\left|Pr\left[S1\right]-Pr\left[S0\right]\right|\leq \frac{\left(q_{h}^{2}+q_{s}^{2}\right)}{2^{\lambda }}+q_{h}\cdot Adv_{Hash}.$$

Game 2 (replace KEM shared key)

In Game 2, the attacker can simulate the decryption process by using the Send query. Since Kyber possesses IND-CCA security, even if *A* is able to decrypt the non-test ciphertext, since it cannot distinguish the actual shared key in the test session from the random string, such a query will not provide any additional advantage regarding the session key SK.

In Game 2, the shared key tsk generated by Kyber-Encap is replaced with a random string of equal length. If the attacker can distinguish Game 2 from Game 1, the algorithm B can be constructed to break Kyber’s IND-CPA security by taking advantage of *A*.

Hence:

(5) $$\left|Pr\left[S2\right]-Pr\left[S1\right]\right|\leq 2\cdot q_{s}\cdot Adv_{Kyber-IND-CPA}.$$

Factor 2 comes from the fact that the protocol is mutual authentication and both parties perform KEM encapsulation/unencapsulation once.

Factor 2 in Formula (5) is strictly derived from the mutual authentication instance. In this instance, both the terminal device and the server invoke Kyber.CCAKEM to negotiate the shared key tsk.

Game 3 (replace MLWE component)

Game 3 aims to map the indistinguishability of the interacting entities of the protocol to the hardness of the Module-LWE (MLWE) problem through a rigorous cryptographic reduction. In this game, the public key components t=As+e that were originally transmitted over an insecure channel and the ciphertext components $v=t⊕r+e_{2}+H\left(ID\|t_{1}\right)$ are replaced with uniformly random vectors.

Construct an algorithm $B^{\prime }$ whose objective is to solve an externally given MLWE problem instance (A,t). The task of $B^{\prime }$ is to determine whether t is a vector that conforms to the MLWE distribution or a uniformly random vector sampled randomly from $R_{q}^{k}$.

$B^{\prime }$ simulates the protocol environment for the attacker A in the following way: $B^{\prime }$ embeds the matrix A from the problem instance into the global parameters of the system as the public key mpk. When A initiates a Send query to start a new session, $B^{\prime }$ no longer generates a temporary secret vector on its own, but directly uses the vector t from the problem instance as the public key component in the protocol response. $B^{\prime }$ constructs the corresponding ciphertext component *v* and authentication mark Auth using the embedded challenge vector t, ensuring that the simulated interaction is statistically consistent with the actual protocol logic.

If the attacker A can distinguish Game 3 from Game 2 with a significant advantage, it implies that A can identify when the vector components change from a valid structure to random noise. At this point, $B^{\prime }$ can utilize the discrimination result of A to solve the MLWE problem: if A believes the input data is genuine, then $B^{\prime }$ outputs “1” (representing an MLWE sample); otherwise, it outputs “0” (representing a random sample).

According to the above reduction process, the difference in success probability between Game 3 and Game 2 is strictly limited within the advantage of solving $q_{s}$ session instances of the MLWE problem:

(6) $$\left|Pr\left[S3\right]-Pr\left[S2\right]\right|\leq 2\cdot q_{s}\cdot Adv_{MLWE}.$$

Game 4 (replace fuzzy commitment keys)

In Game 4, the *k* recovered by the biometric through the fuzzy commitment mechanism is replaced with an independent random 128-bit string.

According to A4, if the attacker can correctly recover *k* without knowing the bio, the probability is at most $Adv_{Fuzzy}+2^{-\lambda }$.

So:

(7) $$\left|Pr\left[S4\right]-Pr\left[S3\right]\right|\leq q_{s}\cdot Adv_{Fuzzy}+\frac{q_{s}}{2^{\lambda }}.$$

Game 5 (replaces final session key)

In Game 5, the final session key $SK=HKDF(tsk||TID^{*}||pk||pk^{\prime })$ is completely replaced with a random 256-bit string. In this case, the attacker does not have any advantage over SK, so: Pr[S5]=1/2.

Game 6 (Perfect Forward Secrecy)

This game evaluates the indistinguishability of the session key SK even if the long-term master secret key msk is compromised. In this game, the adversary A is allowed to issue a Corrupt(CS) query to obtain msk after the test session $\Gamma_{i}^{P}$ has reached the Accepted state.

Reduction Argument: The security of SK in this scenario reduces to the hardness of the MLWE problem associated with the ephemeral keys pk and $pk^{\prime }$. Since the terminal and server erase the ephemeral random vectors $s_{i},e_{i},s_{j},e_{j}$ and the intermediate material tsk immediately after session establishment, the adversary must derive SK solely from the captured transcripts and the leaked msk.

However, SK is derived via $HKDF(tsk,salt=TID^{*}\|pk\|pk^{\prime })$. Even if msk allows the reconstruction of tsk from intercepted $u_{j}$, the entropy of SK is still protected by the ephemeral MLWE samples pk and $pk^{\prime }$. Therefore:

(8) $$\left|Pr\left[S6\right]-Pr\left[S5\right]\right|\leq 2\cdot q_{s}\cdot Adv_{MLWE}.$$

This demonstrates that breaking the forward secrecy of the protocol is at least as hard as solving the MLWE problem.

Summing up the differences across games, we get:

(9) $$\begin{array}{c} \mathrm{Adv}\text{\_}\mathrm{ROR}(A)⩽2\;q\text{\_}s\;\mathrm{Adv}\text{\_}\mathrm{Kyber}\text{-}\mathrm{IND}\text{-}\mathrm{CPA}+2\;q\text{\_}s\;\mathrm{Adv}\text{\_}\mathrm{MLWE}\\ \;\;+q\text{\_}s\;\mathrm{Adv}\text{\_}\mathrm{Hash}+q\text{\_}s\;\mathrm{Adv}\text{\_}\mathrm{Fuzzy}\\ \;\;+\frac{q_{h}^{2}+q_{s}^{2}}{2^{\lambda }}. \end{array}$$

Therefore, under the Assumptions A1–A4 and when the security parameter λ is sufficiently large, the attacker’s advantage is a negligible quantity, and the protocol satisfies the indistinguishability of session keys under the ROR model [30]. This completes the proof.

In conclusion, the absolute advantage $Adv_{ROR}\left(A\right)$ of an adversary in the Real-or-Random model is formally bounded by the sum of advantages in solving the underlying cryptographic primitives. The sequence of game transitions, particularly the transition from Game 2 to Game 3, establishes a rigorous cryptographic reduction. Through the construction of a simulator $B^{\prime }$ that embeds external challenge instances into the protocol execution, we demonstrate that any polynomial-time adversary A capable of distinguishing session transcripts from random strings must implicitly solve the MLWE problem with a non-negligible advantage. Since the MLWE problem is assumed to be computationally hard even for quantum adversaries, the session key SK is provably indistinguishable from a random string in the ROR model. This reduction ensures that the security of the proposed protocol is strictly anchored in the hardness of the underlying Module-LWE assumption.

### 5.2. Informal Analysis

#### 5.2.1. Resistance to Man-in-the-Middle Attacks

This scheme tightly binds the identity credentials, session random quantities, and handshake transcripts through a hash function, ensuring that mutual authentication can be successfully completed and the shared session key SK can be derived only when the terminal $TD_{i}$ and the server $CS_{j}$ simultaneously possess their respective long-term credentials and user secrets (such as ID, PWD, and private keys $s_{i}$, $s_{j}$). From a quantitative security perspective, the strength of the association between the device identifier and the handshake records is guaranteed by the combined security strength of SHA3-256 and Kyber512; if the attacker fails to obtain the corresponding secret values, the probability of successfully forging an authentic message is strictly limited within the range of $2^{-128}$, which is comparable to the security level of a symmetric encryption scheme with 128-bit security (such as AES-128). Since the negotiation of the session key entirely relies on the public key construction that satisfies the MLWE assumption, and breaking such underlying cryptographic primitives is considered computationally infeasible, this scheme can effectively resist man-in-the-middle attacks.

#### 5.2.2. Resistance to Replay Attacks

In this scheme, replay attacks are doubly suppressed by “time threshold + unique random seed”. Firstly, the terminal and the server verify the timeliness of the message through a predefined detection window Δt = 2000 ms. Regarding the possible clock drift between medical IoT devices, the protocol assumes that the entities synchronize periodically via Network Time Protocol (NTP) and introduces a 100 ms protection bandwidth to tolerate minor deviations. Any message that satisfies $\left|t2-t1\right|>\Delta t$ will be directly rejected by the system.

Secondly, even if the adversary attempts to re-inject the old message within the valid time window, since the random seeds ($seed\_t_{i},seed\_r_{i}$) for each round of interaction are randomly generated by the sender and then deterministically expanded by SHAKE-128, replaying the old seeds will cause the server to derive v’ that does not match the original session content, thereby triggering the verification failure of $Auth^{*}\neq Auth$. Moreover, during the reconstruction process, the generation of each polynomial coefficient is bound to a unique context index. This domain-separation technique is mathematically equivalent to independent sampling from a random oracle, ensuring that the seed compression does not introduce exploitable correlations or reduce the entropy of the MLWE instance. This design not only strictly limits the probability of seed conflicts that would enable a replay attack to $2^{-256}$ within the range, but also achieves an ideal and rigorous balance between bandwidth optimization and security by blocking statistical attacks between vector components.

#### 5.2.3. Forward Security

In this scheme, the session key SK is not directly derived from the long-term key msk, but is generated through the key derivation function HKDF from the temporary key material. Specifically, HKDF binds the temporary shared key tsk with the instantaneous public keys pk and $pk^{\prime }$ to derive the final session key SK. Therefore, the security of SK not only depends on the long-term key system, but is also deeply tied to the independently generated temporary random seeds ($seed\_t_{i},seed\_r_{i}$) and the corresponding temporary private key vectors for each session. From a formal perspective, let A be an attacker who can obtain the long-term key msk through post-session tampering queries. To recover the historical session key SK, A must either reverse HKDF or solve the corresponding MLWE instances for the temporary vectors $s_{j}$ and $e_{j}$. However, under the MLWE assumption, the temporary public keys pk and $pk^{\prime }$ generated by these vectors are computationally indistinguishable from uniformly random vectors. Therefore, even if the msk is leaked in the future, the attacker cannot gain any non-negligible advantage in distinguishing the real session key SK from the random string. Moreover, after the authentication process is completed, the terminal and the server will immediately destroy all temporary random seeds, temporary private key vectors, and intermediate key materials tsk from memory. Since these transient random states cannot be restored after the session ends, even if the attacker obtains the long-term private key msk or the user password later, they cannot perform retroactive decryption on the intercepted historical ciphertext because they cannot reproduce the instantaneous random state required to generate SK. Therefore, the security of past sessions will not be affected by the future leakage of long-term credentials, thus ensuring that the protocol satisfies perfect forward secrecy.

#### 5.2.4. Resistance to Offline Dictionary Attacks

The three-factor mechanism effectively blocks offline password guessing in this scheme. Even if the attacker obtains the triplet $\left\{V_{2},h,TID^{\prime }\right\}$ stored locally on the terminal, since the terminal does not persistently store the fuzzy commitment C and the intermediate key k, the attacker lacks the biometric information and random mask needed to reconstruct k, and thus cannot restore k through Hamming decoding or other methods to verify h = H(k). At the same time, the hash value $H\left(ID||pwd||C||mpk\right)$ used for offline comparison depends on the password and the commitment C, and neither of them can be obtained in the offline environment. Therefore, there is no criterion available for verifying the guessing result. The final verification of the password can only be completed in the online two-way challenge-response process. The server only accepts the request after receiving the legitimate authentication message generated by the immediate seed and timestamp, and can constrain the guessing behavior within the controlled online channel through failure count limitation or backoff mechanism, thereby eliminating the possibility of offline dictionary attacks. Moreover, the security of the scheme also relies on the 128-bit entropy provided by the randomly generated key k. Even if the attacker attempts to exhaust the entire key space, with a hash calculation taking approximately 0.15 ms per time, traversing $2^{128}$ possibilities still requires approximately $5.1\times 10^{27}$ years, which is computationally infeasible. Therefore, it can effectively resist brute-force attacks.

To further enhance the “multi-factor” security, this protocol can explore the integration of Physical Unclonable Function (PUF) in the future. Specifically, a PUF based on an accelerometer can be utilized to extract a unique hardware fingerprint from the manufacturing deviations of the terminal motion sensor [31]. This hardware-level security factor, combined with the current Kyber software authentication and biometric blur commitment, will provide a deeper level of defense, enabling the solution to have stronger resilience in resisting device cloning and complex offline credential analysis.

#### 5.2.5. Resistance to Template Inversion Attacks

This scheme combines fuzzy commitment with lightweight Hamming error correction for protecting biological templates [32,33]. The system randomly generates a 128-bit key (k), which is encoded using Hamming to obtain the codeword ($c=Encode\left(k\right)\in {\left\{0,1\right\}}^{256}$), and calculates the auxiliary data (δ=c⊕bio) and the check value (h = H(k)). Since the codeword (c) is derived from an independent random key rather than the original biological template, the terminal only stores the XOR result of the template and the random codeword as well as the check value. Even if the attacker obtains (δ) or (h), they cannot reverse-engineer (bio) or recover (k) and (c) from (h). The fuzzy commitment scheme supports a maximum bit error rate of approximately 17%, enabling legitimate users to stably recover the key in the presence of noise while ensuring the irreversibility of the template. Recovering the original biological template from the stored data requires approximately ($2^{128}$) attempts computationally, which is practically infeasible. Although the key (k) remains static throughout the user’s lifecycle to ensure the recoverability of biological features, the protocol introduces an independently generated random seed in each session to ensure session freshness, and can use independent keys (k) or offsets (δ) between different service providers, effectively blocking cross-system association attacks. This maintains the stability of authentication while enhancing the security and privacy protection level of the template.

#### 5.2.6. Anti-Quantum Attack

This scheme achieves effective defense against quantum attacks by replacing the traditional discrete logarithm system with cryptographic primitives based on the Learning With Errors (MLWE) problem. Compared to the Elliptic Curve Cryptography (ECC) scheme, which is vulnerable to the Shor algorithm under large-scale quantum computing conditions and has a complexity of ($O(\log^{3}n)$), the protocol constructed based on the difficult problem of lattices typically requires sub-exponential computational resources to be broken, significantly increasing the attack cost. According to the NIST post-quantum cryptography standard assessment, the Kyber-512 adopted has a Core-SVP complexity of approximately ($2^{118}$) operations, corresponding to NIST security level 1, providing a stable quantum security margin and ensuring the long-term security of the protocol in the post-quantum environment. At the same time, considering the quadratic acceleration effect of the Grover search algorithm on symmetric cryptographic primitives [34], the scheme using the SHA-256 hash function can still maintain a quantum pre-image security strength of approximately ($2^{128}$) level, thereby achieving a balanced design between asymmetric and symmetric mechanisms, and providing overall consistent and sufficient quantum security guarantees for the Medical Internet of Things environment.

#### 5.2.7. Privacy Compliance and GDPR Adherence

To address the increasing regulatory requirements for sensitive medical data, our protocol is designed according to the “Privacy by Design” principle, ensuring alignment with international frameworks such as the General Data Protection Regulation (GDPR). Specifically, the scheme implements data minimization by ensuring that no raw biometric images or physiological traits are stored on the cloud server (CS). Instead, by employing a fuzzy commitment scheme combined with one-way cryptographic hash functions, the server only retains non-invertible biometric templates. This ensures that even in the event of a complete database compromise, the original biometric information cannot be reconstructed from the stored credentials. Furthermore, the use of dynamic pseudonyms ($TID_{i}$) for user identification prevents linkability, thereby fulfilling the transparency and security requirements mandatory for modern medical IoT deployments.

## 6. Performance Analysis

This section presents a performance comparison of the scheme based on three indicators: functional features, computational cost, and communication cost.

In order to conduct a comprehensive evaluation of the proposed solution, we selected four protocols for comparative analysis. These protocols represent the classic and post-quantum paradigms, respectively. The main basis for selection was the technical relevance and the matching of application scenarios. Specifically, the schemes proposed by Hammi et al. [14] and Abdaoui et al. [15] based on elliptic curve encryption are the foundation benchmarks of traditional lightweight security standards in the IoT environment. In contrast, the results based on lattices by Dabra et al. [18] and Chen et al. [16] were included to conduct a direct performance assessment with contemporary post-quantum solutions, which have an architecture foundation similar to the basic architecture of Kyber. Since all the selected studies are specifically targeted at the resource-constrained IoT environment in healthcare, this comparison framework ensures a fair and rigorous assessment of the key trade-offs between computational efficiency, communication overhead, and functional characteristics.

### 6.1. Functional Features

Regarding forward secrecy, resistance to replay attacks, and resistance to quantum attacks, this paper compares the proposed method with related schemes by Dabra et al. (2021) [18], Chen et al. (2024) [16], Hammi et al. (2020) [14], and Abdaoui et al. [15]. The corresponding outcomes are summarized in Table 3.

Table 3 conducts a comparative analysis of different schemes from aspects such as resistance to man-in-the-middle attacks, resistance to quantum attacks, resistance to replay attacks, forward secrecy, resistance to offline dictionary attacks, and resistance to template inversion attacks. The ECC-based Hammi et al. (2020) [14] and Abdaoui et al. [15] schemes cannot resist quantum attacks, and they also lack the ability to resist offline dictionary attacks and template inversion attacks; while the lattice-based Dabra et al. (2021) [18] and Chen et al. (2024) [16] schemes can resist quantum attacks, they still cannot prevent template inversion attacks. In contrast, the scheme proposed in this paper, like other lattice-based protocols [16,18], has the ability to resist quantum attacks. Its quantum security is evaluated based on the NIST Class 1 security standard, and the elliptic curve-based schemes [14,15] will face an almost zero security margin in the large-scale quantum computing environment due to the efficiency of the Shor algorithm.

### 6.2. Computational Overhead

This section focuses on computational costs and compares the proposed solution with several existing ones. This study conducted a benchmark test on a laptop equipped with an Intel i7-12650H CPU and 16 GB RAM. The experiment was carried out under the Ubuntu 22.04 LTS operating system, using the GCC 11.4.0 compiler and enabling the -O3 optimization option for compilation. Elliptic curve operations were implemented based on the MIRACL library, while the components based on Kyber adopted the official NIST reference implementation. Execution time was measured using the high-precision CPU cycle counter cpucycles(), and 10,000 independent trials were performed for each encryption primitive to ensure statistical rigor. Table 4 shows the running time and arithmetic mean of the server-side operations, effectively eliminating the influence of hardware fluctuations.

In response to the stringent resource-constrained hardware requirements of the MIoT environment, we further mapped the instruction-level complexity of the solution to the widely used ARM Cortex-M4 embedded architecture. This analytical method referred to the official benchmark test data of the Kyber algorithm on a 168 MHz microcontroller by the NIST PQC project, ensuring that the evaluation was not solely dependent on high-performance platforms but was based on the architectural limitations of real-world medical sensors. For the elliptic curve-based solution, we set the size of the elliptic curve points to 256 bits.

Table 5 summarizes the comparison results between the presented scheme and existing approaches. It can be seen that the scheme in [15] requires the terminal and the server to perform two point multiplications and 20 inversions each. The scheme in [14] requires the terminal and the server to perform two elliptic curve scalar multiplications, 1 + n isogeny mappings, 1 + n HMAC-SHA256, 1 + n modular inversions, 2(1 + n) modular square roots, 1 RNG (192-bit), and 8(1 + n) 192-bit modular multiplications each, where n is the number of messages sent. The scheme in [18] requires the terminal and the server to perform eight hashes, three samplings, four polynomial multiplications, three polynomial additions, one Cha() operation, and two Mod operations each. The scheme in [16] requires the server and the terminal to perform five hashes, two samplings, one polynomial addition, six polynomial multiplications, two Rec operations, and one Helprec operation each. In the proposed scheme, the terminal needs to perform nine hashes, two samplings, two polynomial multiplications, two polynomial additions, two compressions, and two decompressions, while the server needs to perform four hashes, three samplings, three polynomial multiplications, two polynomial additions, two compressions, and two decompressions.

**Figure 5:**
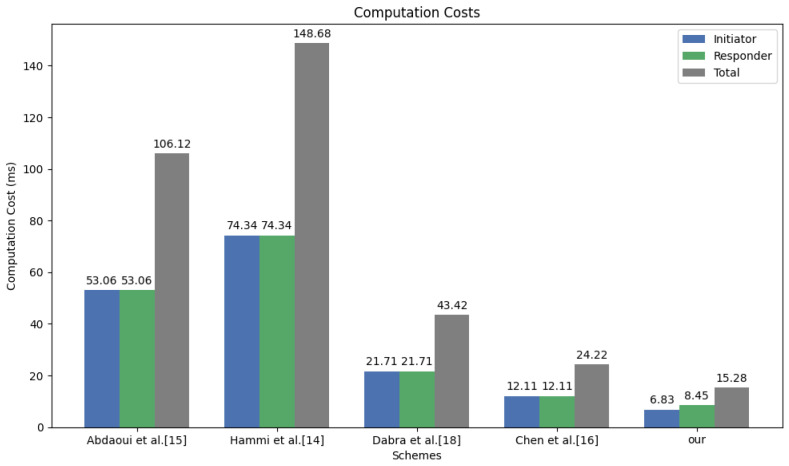
Comparison of computational overhead of various schemes.

To ensure the fairness and rigor of the performance evaluation, and to enhance the transparency and verifiability of the claimed efficiency, we adopted a standardized benchmark testing framework to uniformly standardize the security levels of all the compared protocols [14,15,16,18]. This ensured that all protocols reached an equivalent security level equivalent to NIST Security Category 1 (128-bit classical security). Thus, a comparative analysis was achieved. It should be noted that the original lattice-based schemes proposed in [16,18] were based on different underlying parameter settings. If the original parameters were directly used for comparison, it might lead to inconsistent security margins. Therefore, during the implementation process, we reconfigured the parameters in [16,18] to be consistent with the Kyber512 parameter set (k = 2, n = 256, q = 3329). Meanwhile, the elliptic curve-based schemes in [14,15] used the NIST P-256 curve to correspond to the same security level. Under this unified setting, all protocols were evaluated under the same NIST Security Category 1, ensuring that the performance differences mainly reflect the protocol structure and design efficiency rather than the differences in security margins. Secondly, the total computational overhead shown in Figure 5 is based on the arithmetic accumulation of the time consumed by each atomic operation in Table 3, aiming to uniformly benchmark the inherent efficiency of each scheme objectively under the benchmark (laptop test platform). Based on this, we introduce the aforementioned 100-fold performance scaling factor to map the benchmark test results on the desktop platform to the resource-constrained terminal hardware environment, thereby evaluating the feasibility of the solution in actual MIoT scenarios. This scaling factor is calibrated by comparing the execution cycles of standard SHA-3 hash operations on desktop processors and ARM Cortex-M4 platforms. This method is consistent with the benchmark test results in the pqm4 framework. Therefore, this calibration method can more accurately estimate the actual operating performance of each solution on resource-constrained MIoT sensor devices. We quantitatively defined the efficiency improvement η as $\eta =\frac{T_{existing}-T_{proposed}}{T_{existing}}\times 100\%$. Applying this indicator to the experimental data in Figure 5 confirmed that this scheme reduced the computational overhead by 64% compared to [18] and by 36% compared to [16]. This two-stage evaluation method of “benchmark testing + hardware projection” not only enhances the rigor of the research results but also ensures the repeatability of performance evaluation under different hardware scales.

Experimental evaluations have shown that due to the lightweight nature of its core components (such as Kyber-512 and SHA-3), the proposed protocol is highly feasible for MIoT deployment. Referring to previous studies [10,13], the execution cycle of Kyber on the ARM architecture can be optimized to the millisecond level. Even under conservative assumptions (i.e., the performance of the microcontroller is two orders of magnitude lower than that of a desktop CPU), the overall authentication delay at the device end is expected to remain below 500 milliseconds. This delay level is highly compatible with the timing constraints of medical wearable systems, and compared with traditional ECC methods, it demonstrates significant efficiency advantages in low-power and communication-constrained scenarios.

Furthermore, considering the storage space limitations of MIoT devices, we evaluated the memory footprint of the solution based on the standard Kyber implementation under the ARM Cortex-M4 architecture. Referring to the widely recognized pqm4 benchmark test [35], the peak RAM occupancy of this solution at the terminal side is approximately 2.8 KB, and the Flash code volume is approximately 11.5 KB. For typical medical sensors, such as STM32 or nRF52 series, this storage requirement is far below the hardware threshold, demonstrating the resource compatibility of the solution in both static storage and dynamic operation dimensions.

### 6.3. Communication Overhead

This section focuses on the comparative analysis of communication overhead. For the convenience of comparison, we set the password and identity storage as 64-bit binary strings and the timestamp as 32 bits.

The parameters set in this scheme are k = 2, n = 256, and q = 3329. In the initial stage of key negotiation, the terminal sends a request of 32 bits to the server, and then the server returns a 32-byte secret number. Subsequently, the terminal sends 800 bits of data to the server. The specific composition includes a 16-byte compressed seed $seed\_t_{i}$ and $seed\_r_{i}$ for polynomial reconstruction, a 32-byte SHA3-256 identity authentication tag Auth, 4 bytes of timestamp t1, and 32 bytes of P. Additionally, the 512-bit compressed key refers to the internal storage format of the terminal, retaining only 256 bits of the generated seed and 256 bits of the biometric commitment hash (h = H(k)), which significantly reduces the memory burden compared to the 800-byte standard Kyber public key, while maintaining post-quantum security strength.

**Figure 6:**
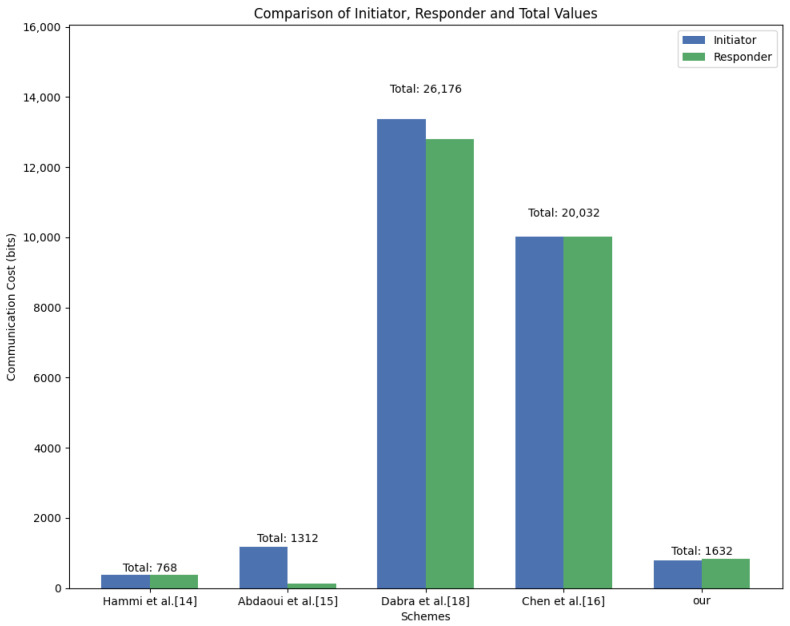
Comparison of communication overhead of various schemes.

When compared with the existing studies, the scheme proposed in this paper demonstrates a significant efficiency advantage. As shown in Table 4, for the lattice cipher scheme [18] (n = 512, q = 7,557,773), the data sent bidirectionally in one round of communication is 13,376 bits and 12,800 bits, respectively; for the scheme [16] (k = 3, q = 211, n = 256), the bidirectional sending volumes are both 10,016 bits. The public key and ciphertext of the standard Kyber512 usually require more than 12,500 bits of bandwidth. Through the compression mechanism of this scheme, we only retain the core seed of 256 bits and the biometric hash value h=H(k) of 256 bits as the security credential. This results in the effective payload of a single authentication communication being only 800 bits, and the overall overhead being 1632 bits. Figure 6 visually and intuitively reflects the differences in communication costs among different schemes. It can be seen that due to the ECC-based schemes (such as the schemes of Hammi et al. [14] and scheme [15]) benefiting from extremely mature hardware optimization support and smaller original parameter quantities, their communication costs still have certain advantages. However, by comparing with other similar lattice schemes, it can be seen that this paper’s scheme has reduced the communication cost by more than 90%. Although the cost is slightly higher compared to the ECC schemes, in terms of security, lattice-based schemes have significant quantum resistance and provide higher long-term security guarantees. With the integration of modern MIoT devices with hardware acceleration engines for primitives such as SHA-3, this scheme will have a more competitive actual deployment performance while maintaining an extremely low load of 800 bits.

Figure 6 visually reflects the differences in communication overhead between different schemes. It can be seen that lattice-based schemes have significantly higher communication overhead than ECC-based schemes. Compared with the other two lattice-based schemes, the proposed scheme effectively reduces the communication cost. While the communication overhead of the scheme exceeds that of ECC-based solutions, lattice-based approaches offer strong resistance to quantum attacks and provide enhanced security. Moreover, ongoing advances in communication technologies are expected to further reduce this overhead.

In extremely energy-constrained wearable environments, duty-cycled communication paradigms such as concurrent low-power listening can significantly reduce energy consumption. The proposed authentication protocol operates independently of the underlying communication scheduling mechanism and can therefore be integrated with such low-power networking strategies.

### 6.4. Energy Consumption

To evaluate the sustainability of the proposed scheme for battery-powered MIoT terminals, we conduct an energy consumption estimation based on the measured computational and communication costs. The total energy expenditure (*E*) is derived using the formula E=V·I·t, where *V* and *I* represent the operating voltage and current of a typical MIoT processor (e.g., ARM Cortex-M4 at 3.3 V and 15 mA), and *t* is the execution time.

Based on the terminal-side processing time and the 800-bit communication overhead, the estimated energy consumption per authentication session is approximately 1.50 mJ. This low energy footprint ensures that the device can sustain thousands of authentication cycles on a standard coin-cell battery, confirming the protocol’s suitability for long-term deployment in medical sensors and implantable devices.

## 7. Conclusions

For the Medical Internet of Things (MIoT) scenario, this paper proposes a lightweight Kyber identity authentication scheme. By introducing the post-quantum key encapsulation mechanism and combining it with the fuzzy commitment technology, while supporting two-way authentication and secure session key negotiation, this scheme significantly enhances the privacy protection capability of users’ biometric data. The proposed scheme is formally proved to satisfy the indistinguishability of session keys in the Real-or-Random (RoR) security model. Through informal security analysis, its robustness in defending replay attacks, man-in-the-middle attacks, offline dictionary attacks, and template inversion attacks is verified. The performance evaluation results show that this scheme outperforms existing lattice-based cryptographic schemes in terms of computational complexity and communication cost, and has significant anti-quantum attack performance and practicality. Moreover, the security channel constructed by this scheme can also provide the necessary trust framework for digital twin models and cross-device transfer learning, ensuring the legality and completeness of medical diagnosis data in complex interaction environments. This balance of security and efficiency provides a practical reference solution for the security authentication mechanism and advanced applications of the Medical Internet of Things in the post-quantum era.

In practical MIoT deployments, the performance of authentication schemes is also influenced by communication and networking mechanisms. Efficient anti-collision techniques can reduce authentication latency when multiple devices attempt to access the network simultaneously. For example, distributed parallel particle swarm optimization-based reader anti-collision methods have been shown to significantly improve identification efficiency in large-scale IoT systems.

Moreover, energy efficiency remains a critical concern for wearable devices with limited battery capacity. Communication paradigms such as concurrent low-power listening enable duty-cycled operation, which can significantly reduce energy consumption while maintaining device responsiveness.

Beyond cryptographic authentication, complementary security techniques may further strengthen the overall system. Physical-layer approaches such as NOMA-assisted semi-grant-free transmission can enhance communication security and reliability, while hardware-based primitives such as Physical Unclonable Functions (PUFs) may provide unique device fingerprints to reinforce device identity verification. In addition, preprocessing techniques such as outlier detection can improve the reliability of biometric features used in fuzzy commitment mechanisms.

The proposed authentication framework can also support emerging healthcare applications that require secure data exchange, such as digital twin-based medical systems and cross-device healthcare analytics. Furthermore, privacy-preserving techniques such as sequential multi-signer ring signatures can enhance anonymity when authenticated devices share medical data with cloud platforms. Finally, network-level optimizations, including energy-efficient multipath routing schemes, can work together with the proposed authentication protocol to improve the scalability and longevity of large-scale IoMT deployments.

## Figures and Tables

**Table 1:** Comparison of related works in IoMT authentication schemes.

Category	Representative Tech.	Core Strengths (Pros)	Main Limitations (Cons)
Traditional Lightweight	ECC, Hash, XOR-based schemes	Short keys, high computational efficiency, and low implementation cost.	Vulnerable to quantum attacks; performance drops on constrained devices; lacks forward secrecy.
PQC Foundations	RLWE/MLWE (e.g., LBA-PAKE)	Quantum-resistant; supports anonymity and forward secrecy; formal ROR proofs.	High communication overhead (>10k bits); complex server-side processing; signal leakage risks.
Error Reconciliation	Signal functions, Voronoi cells	Reduces data transmission during key agreement.	Requires additional polynomial computation and increases implementation complexity.
Biometric Protection	Hamming code + Fuzzy Commitment	Avoids raw biometric template storage and improves privacy protection.	Error correction decoding may introduce additional processing latency.
Protocol Optimization	Round reduction, Batch verification	Reduces interaction rounds and improves authentication efficiency.	Requires strict time synchronization and may introduce replay attack risks.

**Table 2:** The notation table of the proposed scheme.

Notation	Definition	Notation	Definition
k,q,m,n	Safety parameters	msk	Master secret key
${\mathrm{TD}}_{i}$	Terminal device *i*	mpk	Master public key
${\mathrm{CS}}_{j}$	Cloud server *j*	⊕	XOR operation
${\mathrm{ID}}_{i}$	Identity of ${\mathrm{TD}}_{i}$	H(·)	Hash function
${\mathrm{ID}}_{j}$	Identity of ${\mathrm{CS}}_{j}$	$t_{i}$	Timestamp
bio	Binary vector of biological features	KDF	Key derivation function
$seed_{t_{i}},seed_{r_{i}}$	Compression seeds for vector reconstruction	SK	Session key
Auth	Authentication tag	XOF(·)	Extensible output function

**Table 3:** Comparison between the functional characteristics of the various schemes.

Scheme	Resistance to Man-in-the-Middle Attack	Resistance to Quantum Attack	Resistance to Replay Attack	Forward Secrecy	Resistance to Offline Dictionary Attack	Resistance to Template Inversion Attack
[15]	✓	×	✓	✓	×	×
[14]	✓	×	✓	✓	×	×
[18]	✓	✓	✓	✓	✓	×
[16]	✓	✓	✓	✓	✓	×
Our	✓	✓	✓	✓	✓	✓

**Table 4:** Each operation and its time spent.

SHA3-256	0.15	Polynomial multiplication	0.49
SHA-512	0.16	Polynomial addition	0.37
Generation matrix	1.96	Rec	0.82
Random sampling (Kyber)	1.88	Helprec	1.08
Random sampling	2.64	Isogeny (n = 5)	0.14
ECC scalar multiplication	35.76	Modular square root	0.08
Elliptic curve dot product	24.63	Cha()	0.89
Modular inverse of a large integer	0.02	Mod	0.5

**Table 5:** Comparison of the operation of each scheme.

Scheme	Initiator	Responder
[15]	carry out 2 times of point multiplication and 20 times of inverse element	carry out 2 times of point multiplication and 20 times of inverse element
[14]	twice elliptic curve scalar multiplication, 1 + n times isogeny mapping, 1 + n times HMAC-SHA256, 1 + n times modular inverse, 2(1 + n) times modular square root, 1 time RNG (192-bit), and 8(1 + n) times 192-bit modular multiplication	twice elliptic curve scalar multiplication, 1 + n times isogeny mapping, 1 + n times HMAC-SHA256, 1 + n times modular inverse, 2(1 + n) times modular square root, 1 time RNG (192-bit), and 8(1 + n) times 192-bit modular multiplication
[18]	8 hashing times 3 sampling times 4 polynomial multiplication times 3 polynomial addition times 2 polynomial addition times 1 cha() operation times 2 mod operations	8 hashing times 3 sampling times 4 polynomial multiplication times 3 polynomial addition times 2 polynomial addition times 1 cha() operation times 2 mod operations
[16]	5 hashes, 2 sampling, 1 polynomial addition, 6 polynomial multiplications, 2 Rec operations and 1 helprec operation	5 hashes, 2 sampling, 1 polynomial addition, 6 polynomial multiplications, 2 Rec operations and 1 helprec operation
Our	9 hashing 2 sampling 2 polynomial multiplication 2 polynomial addition 2 compression 2 decompression	4 hashing 3 sampling 3 polynomial multiplication 2 polynomial addition 2 compression 2 decompression

## Data Availability

Data are contained within the article.
